# Perception accuracy, biases and path dependency in longitudinal social networks

**DOI:** 10.1371/journal.pone.0218607

**Published:** 2019-06-18

**Authors:** Güneş Ertan, Michael D. Siciliano, Deniz Yenigün

**Affiliations:** 1 Department of International Relations, Koç University, Istanbul, Turkey; 2 Department of Public Administration, University of Illinois at Chicago, Chicago, IL, United States of America; 3 Department of Industrial Engineering, Istanbul Bilgi University, Istanbul, Turkey; Middlesex University, UNITED KINGDOM

## Abstract

Most studies on perceptions of social structures in organizations rely on cross-sectional evidence and lack a longitudinal perspective. In order to address this gap, we collected whole network perception data at three time points from a cohort of MBA students. First, we asked whether or not individuals become more accurate in their perception of the network over time. We found no significant increase in accuracy. Second, we examined one’s perception of his or her own direct ties and found a consistent tendency to inflate incoming friendship ties, confirming existing studies. However, we find that individuals were quite capable of recognizing the broader dynamics of social hierarchy (i.e., whether they were becoming more or less popular) even as they became no more accurate in understanding either the overall networks or their own ego-net. Third, we explored possible explanations for the persistence of perception errors and showed that most of the errors at time point two and time point three were due to a failure to update previous perception decisions. Finally, we shifted the analysis from accuracy at a given time point and considered the narrative arc of dyadic relations. Our findings suggest that stable dyads across time are more likely to be accurately perceived whereas other types of dyads are poorly tracked. We conclude by presenting possible research questions for future studies to further our understanding of the temporal aspects of network perception.

## Introduction

Extant research indicates that individuals’ perceptions of social relations they are embedded in have consequences on a variety of important outcomes such as power, performance, reputation, leadership, enhanced team performance, coordination and innovation [1–6]. Due to the importance of network perception, a sub-discipline of organizational social network research, known as cognitive social structures (CSS), focuses on how individuals perceive and represent the networks that surround them. However, despite the dynamic nature of social networks, an analysis of perceptions of informal networks at multiple time points has been missing from the literature. This is mostly due to the heavy burden placed on respondents of CSS surveys and the reluctance of organizations and individuals to participate in such time consuming studies at multiple points in time. Consequently, existing network perception studies using whole network designs are based on perceptions of network structure at a single point in time. Therefore, we have very limited knowledge on how perceptions evolve over time. In this paper, we explore patterns of change in perceptions of networks at three time points and provide tools and concepts to encourage other network researchers to study longitudinal network perception data. In the following sections, we exploit a novel data set that captures the evolution of network perception, and show that despite common sense expectations, accuracy in perceiving social structure does not improve over time. Our findings do confirm existing literature on the tendency for respondents to inflate their own centrality [7]. However, despite consistent overestimation of how central one is in the network, we find that the individuals are fairly accurate in terms of recognizing changing trends (i.e., becoming more or less popular) in their informal ties over time. Moreover, we develop a new time-based schema that individuals may rely on to shape their perceptions of their social surroundings. We illustrate that the majority of perception errors are due to individuals’ inability to update their prior perceptions of ties rather than making erroneous updates. Finally, we trace the history of a given tie’s status over time and show that stable relations are more likely to be accurately perceived in comparison to unstable, emerging or dissolving relations.

## Antecedents and consequences of cognitive social structures

Network perception research is a subfield of social network analysis and is mainly concerned with understanding (i) the factors that shape individuals’ perceptions of not only their immediate ties, but also *ties among all other actors* in a bounded network, and (ii) the consequences of these perceptions at both the individual and organizational level [8]. According to Krackhardt [1], studying perceptions matters because people’s beliefs about their own and others’ relations shape their decisions and behaviors. Krackhardt’s ideas have been confirmed and expanded by a range of studies [5, 8]. For example, individuals who are perceived as central actors or perceived to be friends with prominent actors are also perceived as high performers and charismatic, regardless of their actual or “true” position in the network [3]. Likewise, when a person perceives herself to occupy structurally similar positions as others in the network, she is more likely to behave similarly, independent of the actual presence of structural equivalence [9, 10]. This line of research also demonstrates a number of positive outcomes associated with the accuracy of network cognition, such as being perceived as powerful and generous [2, 11]. Moreover, the well-documented benefits of brokerage positions are shown to be available to individuals that can accurately perceive the structural holes in their ego networks [12–14]. In addition to the individual outcomes, studies show that when network perception at the aggregate level reflects actual relations, there are certain positive organizational outcomes, such as innovation, less work duplication, enhanced team performance and better knowledge management [6].

Given the important consequences of network perception for individuals and organizations, another line of research on cognitive social structures concentrates on understanding the formation processes of one’s perceptions. Studies show that central actors and individuals with a low power status tend to have more accurate perceptions of their structural surroundings [1, 15]. Individual traits, such as self-monitoring and a need for high achievement have been shown to be good predictors of perception accuracy [11, 16]. Recent studies have examined the role of gender in shaping cognitive social structures. Brashear et al. [17] and Neal et al. [18] find that women are significantly better at perceiving their network but do not test for possible mechanisms. A number of recent experimental studies show that women tend to be more prosocial, altruistic, and honest in comparison to men due to social roles and norms [19–21]. To what extent these traits can explain network cognition remains an important open question.

## Cognitive social structures across time

Understanding the temporal aspects of network perceptions matters a great deal, not only theoretically but also as a practical matter for managers and organizational leaders. We know that there are various positive organizational outcomes, such as enhanced team performance, coordination and knowledge management and transfer that accrue to organizations when employees have accurate perceptions of their informal social structures in organizational settings [6]. Therefore, strategies and interventions aimed at increasing the visibility of informal ties in organizations can be devised more effectively when the trajectories of network perceptions and time dependent systematic errors are reflected in the design process.

### Perceptions over time

In the following sections we first examine the extent to which individuals tend to “*learn*”the social structures that surround them. Based on available longitudinal interpersonal network data, we know that some friendships are stable while others fluctuate [22]. However, our knowledge is considerably limited with regard to the perception patterns of these relations over time. While there is some evidence suggesting perception accuracy improves over time when the network size is smaller [18], Kilduff and Krackhardt [5] argue individuals’ network perceptions can be stable or change over time based on changes in schemas used to encode the social ties.

Next, we shift the focus from how accurate individuals are in perceiving the status of ties between other members of the network to one’s ability to perceive his or her own relationships. Previous studies have shown that individuals’ network perceptions are very much egocentric, leading individuals to systematically perceive themselves to be more central than they actually are [7, 23]. Self-enhancing biases, as in the case of inflated ego-net perception, are frequently explained by people’s innate desire to perceive themselves positively. Self-enhancement not only helps with maintaining self-esteem, but may also be critical for mental health, level of happiness and capacity for creative and productive work [24, 25]. Krebs and Denton [26] suggest that self-serving biases may also have an adaptive function by generating self-fulfilling prophecies. Therefore, we may expect people to claim to have ties with peers that are not confirmed. However, we do not know whether and to what extent learning processes are involved in correcting this bias over time [27].

In addition to assessing changes in one’s overall accuracy in perceiving the whole network, we also investigate perception errors within ego networks over time. While existing research indicates a tendency to inflate one’s own popularity, some studies suggest that individuals’ mental representations of the social structures that surround them are not totally independent from the actual network structures. For example, Zuckerman and Jost [28] show that respondents’ perceived popularity is significantly correlated with true popularity and they conclude that “the presence of bias does not indicate no correspondence between a student’s actual popularity relative to others and his or her perception of reality” (p. 216). Evidence from evolutionary biology and neuroscience also show that individuals are fairly good at perceiving status cues and understand their relative status roles [29]. According to Koski et al. [29], because status hierarchies are relational and dynamic, accurate cognition of these hierarchies is vital for individual adaptation and the functioning of groups. Therefore, we may expect some degree of match between perceived and true changes in the ego-nets of individuals across time, despite a continuing presence of inflated ego-net perception.

### Systematic biases and path dependency

Studies on network cognition show that there are systematic biases that shape individuals’ representations of the social structures that surround them. There is extensive research that shows individuals rely on various schemas such as reciprocity, balance theory, triadic closure and small world principles when recollecting social interactions [30–34].

When considering network cognition errors longitudinally, failure to update prior perceptions may be another important schema. According to the well-established behavioral economics literature, a number of cognitive biases may lead to path dependent judgments that are persistently inaccurate [35]. These include status quo bias, conservatism bias, and confirmation bias. According to the status quo bias, individuals have a strong tendency to stick with the status quo options and conditions. Samuelson and Zeckhauser [36] suggest a number of bounded rationality related mechanisms that may explain the status quo bias, such as loss aversion, anchoring, regret avoidance, and the drive for consistency.

Conservatism bias, on the other hand, suggests that individuals are unlikely to revise their judgments when they encounter new information [37]. Misperception, mis-aggregation, and anchoring (the tendency to stick with the information provided first) are some of the explanations offered for conservatism bias [38]. Since perceptions of social structures are formed on the basis of interactions at discrete time points, we can consider that members of a dynamic social network are consistently provided with “*new information*” or evidence regarding the status of ties around them.

Conservatism bias indicates a reluctance to use new information in updating opinions or positions regardless of the match between the new information and existing beliefs and opinions. In contrast, according to confirmation bias, individuals are more likely to recall, evaluate or search for information that will confirm their existing views [39]. In other words, while conservatism bias indicates a tendency to block or insufficiently weight new information in the updating process, confirmation bias is observed when individuals overweight or misperceive evidence in order to corroborate their prior judgments. The search for consistency and relative ease of processing information that is in line with existing views and opinions are considered the explanatory mechanisms for confirmation bias [40].

Status quo bias, conservatism bias, and confirmation bias suggest that if people perceive two people as friends at an initial time point, they may overly rely on that perception going forward, even in the face of contrary evidence. By considering the temporal aspect of perception errors, we examine the extent to which a time-based heuristic can explain systematic deviations of perceived ties from actual ties. We conceptualize these biases and tendencies to stick with one’s past belief as a form of path dependency.

### The arc of friendship

**Fig 1:**
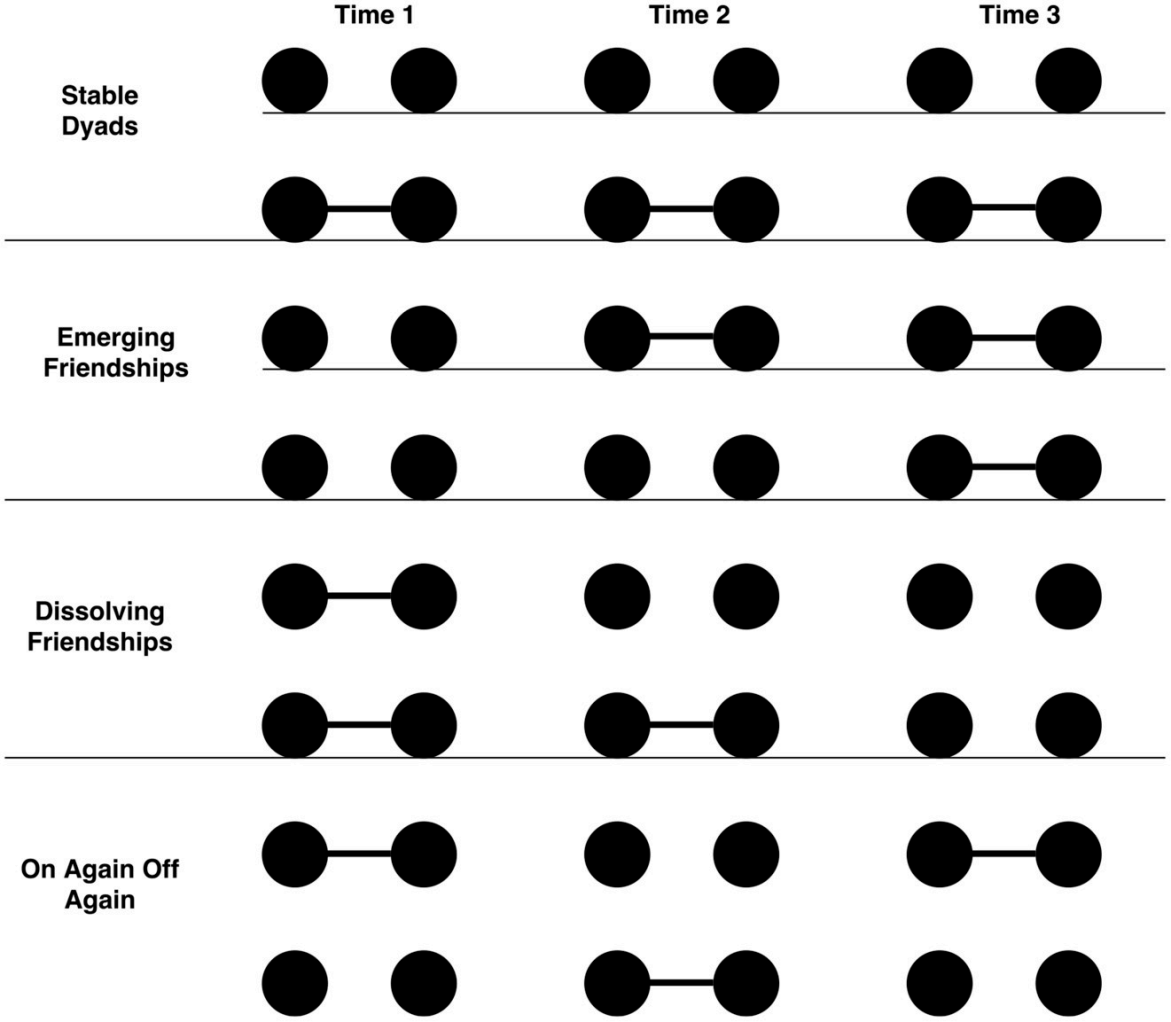
The story arc of friendships.

Models that attempt to predict network structure and formation focus on determining the presence or absence of a tie at a given point in time. However friendships, like other relationships, tend to have a narrative arc that defines their evolution. Some individuals become friends quickly and maintain a stable relationship. Other friendships evolve more slowly or are characterized by an “*on-again, off-again*” pattern. Rather than attempting to examine the accuracy of perception at each time point, we examine the ability of individuals to follow the status of a dyad across time. Assuming data capturing ties at three time points, any given dyad could follow one of eight storylines, as shown in Fig 1.

## Data and methods

We collected data on friendship ties among an incoming MBA cohort of 26 students. Based on their work experience and previous education, none of the students had prior acquaintance with one another. We used a CSS roster design. CSS roster designs are used to study actual social networks in bounded settings. In CSS studies, a network with a size of *N* is represented by a three dimensional array *R*_*i*,*j*,*k*_ (*i*, *j*, *k* = 1, …, *N*), where *i* is the sender, *j* is the receiver, and *k* is the perceiver of the tie. Respondents in CSS designs answer *N*^2^ − *N* possible relations in the network, producing *N* perception matrices that are also known as “*slices*” [1].

Data was collected at a top management school in Turkey (Bilkent University). The school is accredited by the Association to Advance Collegiate Schools of Business (AACSB) International and is consistently included among the best business schools of Eurasia and the Middle East by the Eduniversal ranking program. Students were recruited using an online survey tool. Bilkent University ethics committee approved the study. The meeting number of the approval of the study is 2012092101. Approval was signed by member Prof. Dr. Cemal Yalabik. The data were analyzed anonymously. All 26 of incoming MBA students received the online survey in their school e-mail inboxes. Completion of the survey was optional and no rewards were offered. The consent of the participants were obtained via the online survey as well. Respondents could opt out of answering specific questions and could submit incomplete surveys.

Data was collected at three different time points using a standard roster instrument. The first wave of data was collected in September 2012, during the third week of classes when friendship ties are expected to emerge [5]. The second wave was collected in December 2012, during the fourteenth week of the first semester. The third wave was conducted during the second semester in May 2013, approximately fourteen weeks after the second wave. Response rates showed a decline at the third wave (89 % at Time 1, 85 % at Time 2, and 62 % at Time 3). A student missing at Time 1 may be present at Time 3 or vice versa. Overall there were 17 respondents that completed the all three waves of the study.

Students’ self-reported friendships were measured based on a weighted scale (0-Just a fellow student; 1-Slight friends (acquaintance); 2-Fairly good friends; 3-Close friends; 4-Best friends). Ties reported as a 2 or above were considered as friends in the following analysis. This decision coincides with how the respondents understand friendships. When we construct a network based on individuals’ claims about who sends them a friendship tie (columns of the perception matrices), the density of this network most closely corresponds to the density of the network based on “Fairly good friends” threshold.

Perceptions of ties that existed among the respondent’s classmates were collected using a binary scale. As with other CSS surveys, the respondents were asked to “Consider your fellow student <enter name>. Please indicate on the list below which students <enter name> would consider to be a personal friend.” The respondent could then check all of the names that applied.

Based on the work of Krackhardt [2], for the CSS array at each time point, we constructed the true network using the locally aggregated structures (LAS) intersection rule. Krackhardt [2] argues that the most informed actors concerning the presence or absence of a tie are the two members who constitute the dyad under question. According to LAS-intersection, a tie is considered to be present in the true network if and only if both parties in a dyad report the tie. More specifically, for the directional friendship tie from *i* to *j* to exist, actor *i* had to indicate sending a friendship tie to *j*, and *j* had to indicate receiving a tie from *i*. Therefore, the intersection rule does not force the resulting network structures to be symmetric. This is important because even affective ties like friendship can be asymmetric and should be modeled as such [41]. Construction of the true network based on Butts approach yielded comparable results [42].

While constructing the true network using the LAS intersection rule, the missing students posed a problem, as their responses regarding their own ties were not observed. We address this problem by constructing the true networks for all three waves using the RTM algorithm [43]. Originally developed for estimating networks based on perceptions of a random sample from a bounded social network, the RTM algorithm may conveniently be used to define the LAS intersection based true network in case of missing values. This is due to the fact that the algorithm uses LAS intersection to define the network ties involving the observed individuals, and employs the Receiver Operating Characteristic (ROC) curve to define the network ties for unobserved individuals, balancing the trade offs between omission and commission errors. In case of missing data an alternative way to define the true network would be Krackhartd’s approach [2], however our robustness checks indicate that RTM method performs better.

The correlation between networks in Time 1 and Time 2 is 0.39 and Time 2 and Time 3 is 0.51, indicating a consolidation trend in the true network structure at the time of the last survey. Finally, Table 1 documents the amount of churn that occurred within the network between each of the time points. A significant number of ties were gained and lost at each new observation point as indicated by the Jaccard coefficient.

**Table 1:** Network churn.

	Tie Change				
	0 → 0	1 → 1	0 → 1	1 → 0	Jaccard Coefficient
Time 1 to Time 2	449	83	78	66	0.366
Time 2 to Time 3	464	97	51	64	0.458

## Results

### Perceptions over time

**Fig 2:**
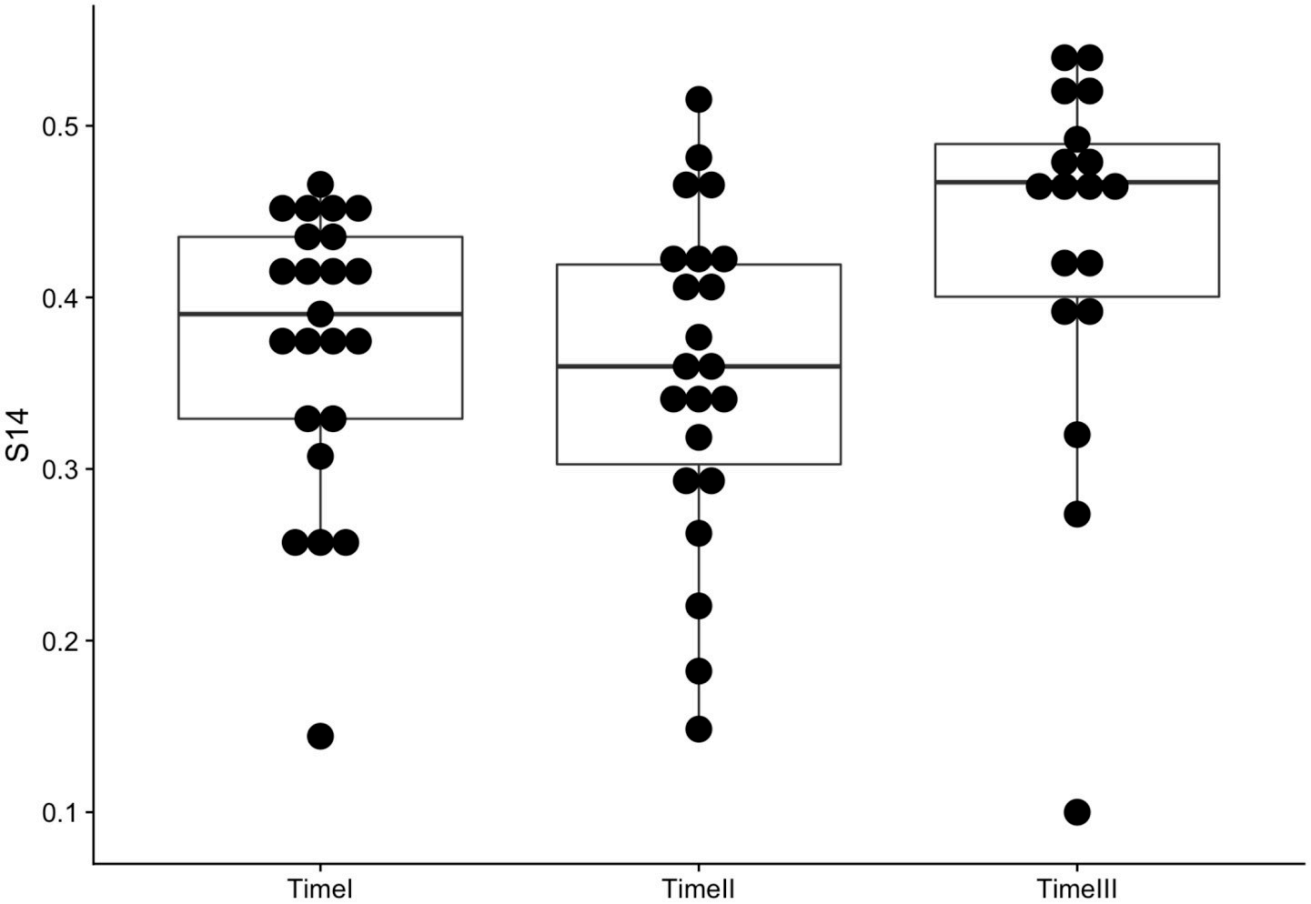
Accuracy over time.

We measured the accuracy of network cognition per individual using *S*_14_ score [44] as recommended by Krackhardt [2]. Accuracy here is defined as the correspondence between an individual’s perception of the network and the true network structure. Fig 2 displays individuals’ accuracy scores over time. Despite a slight increase at Time 3, we do not observe any statistically significant changes overall in individuals’ accuracy scores over time, with averages of 0.37 at Time 1 (SD = 0.084), 0.36 at Time 2 (SD = 0.092), and 0.43 at Time 3 (SD = 0.112). Formal tests (repeated measures ANOVA) failed to show significant differences in average accuracy at different time points (*F*_1,16_ = 3.89, *p* >0.05).

**Fig 3:**
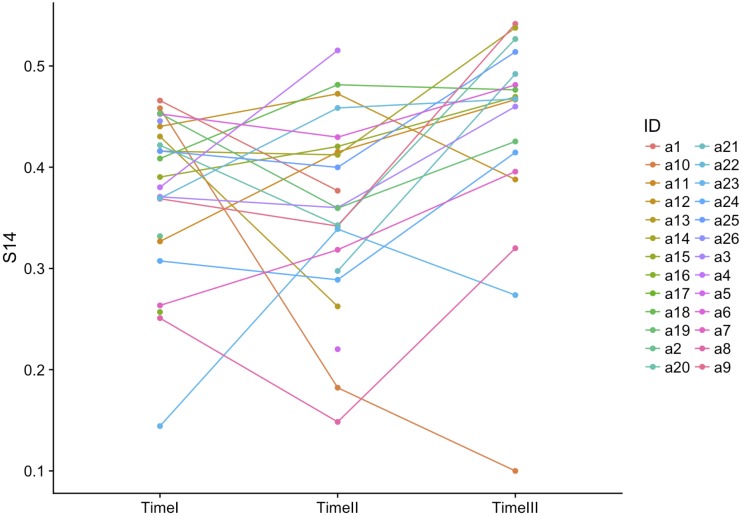
Accuracy over time per student.

Fig 3 displays the trajectory of each student’s accuracy over time. Each line represents a student. No dominant pattern emerges from the figure. Rather, the figure demonstrates the presence of a multitude of trends. Some individuals improved gradually over time, others declined at each time point, and many reversed trends.

### Systematic biases and errors

**Fig 4:**
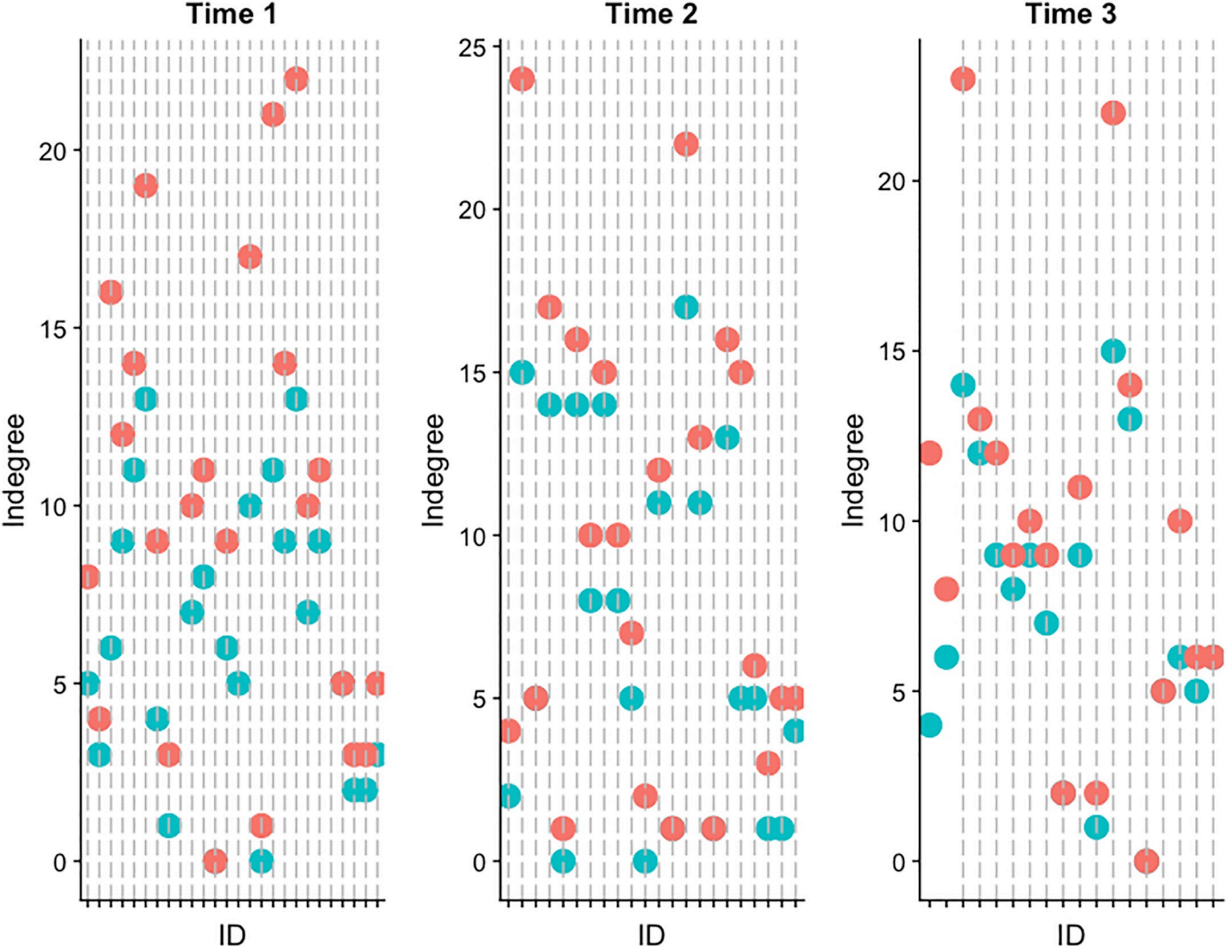
Perceived indegree (red dots) vs. true indegree (green dots).

Next, we look into the fluctuations of some well-known network biases such as inflated indegree centrality. The red dots in Fig 4 denote the reported number of incoming ties and the green ones show actual indegree values (as determined by LAS intersection). We find that individuals tend to exaggerate their incoming ties and that the tendency to overestimate one’s centrality persists across all three waves. However, there is a slight decrease in the average difference between the number of perceived and true indegrees, but it is not statistically significant (Time 1 mean = 3.61, SD = 2.9; Time 2 mean = 2.5, SD = 2.46; and Time 3 mean = 2.38, SD = 2.73; repeated measure ANOVA, *F*_1,16_ = 2.098, *p* > 0.05).

**Fig 5:**
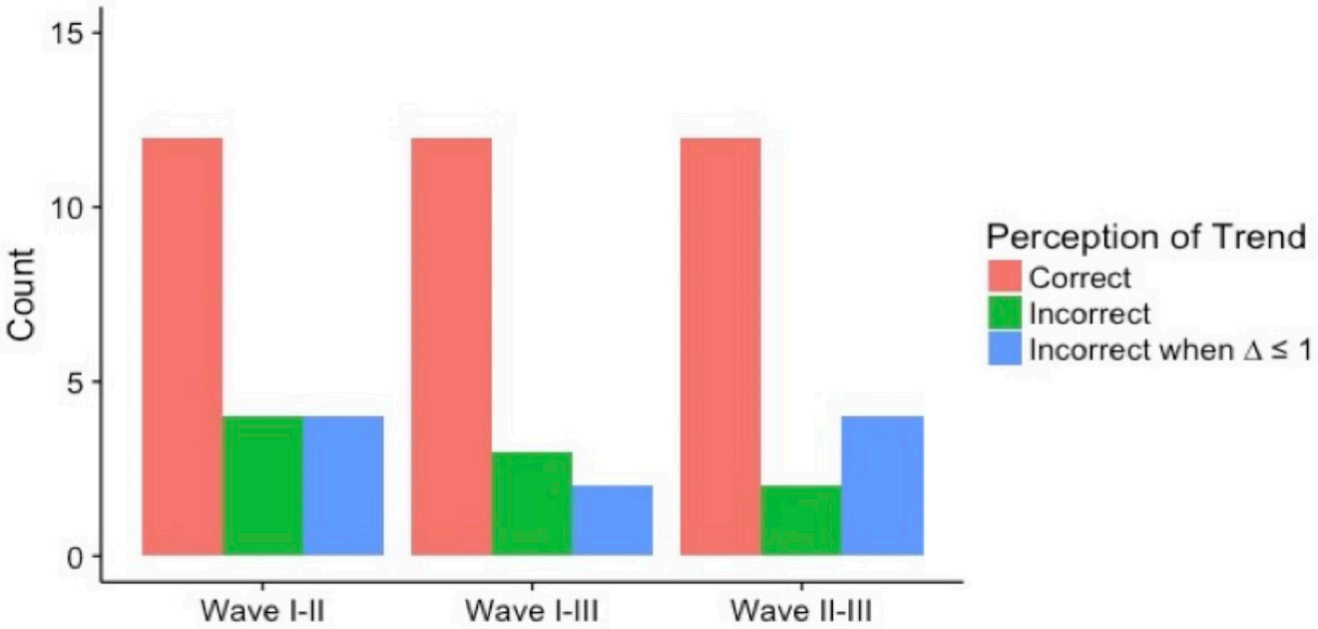
Perception accuracy of trends.

While inflation of indegree seems to be a habitual bias, this does not necessarily mean individuals do not perceive broader changes in their social standing or position. Fig 5 displays the frequency of actors who correctly detected the direction of change in their popularity as measured by indegree centrality. The vast majority of all respondents at each of the three time points correctly perceived whether they were becoming more or less popular. Thus, despite respondents’ continual inflation of their own centrality, they were able to perceive the overall trend in their popularity.

### Path dependency

Humans are prone to cognitive biases that limit their ability to update prior positions or beliefs. Status quo bias, conservatism bias and confirmation bias indicate that individuals are motivated to be attached to their past decisions and hesitant to update those decisions, even in the face of new information. When considering perception errors at Time 2 or Time 3, those errors can arise in one of two ways. First, the respondent may incorrectly perceive the status of a friendship dyad by failing to update their prior beliefs. Here, the respondent may have claimed *i* and *j* were friends at Time 1 and continued with that claim of friendship at Time 2, despite the fact that *i* and *j* were not friends at Time 2. This is an example of path dependency, where individuals tend to stick by their original decision. Second, the respondent may have incorrectly updated their prior beliefs. For example, the respondent may have claimed *i* and *j* were not friends at Time 1 but changed their assessment at Time 2 and incorrectly claim that *i* and *j* are friends. By examining all errors occurring at the second and third time point, we are able to assess the extent to which those errors are the result of failing to update a prior belief or incorrectly updating a prior belief.

**Fig 6:**
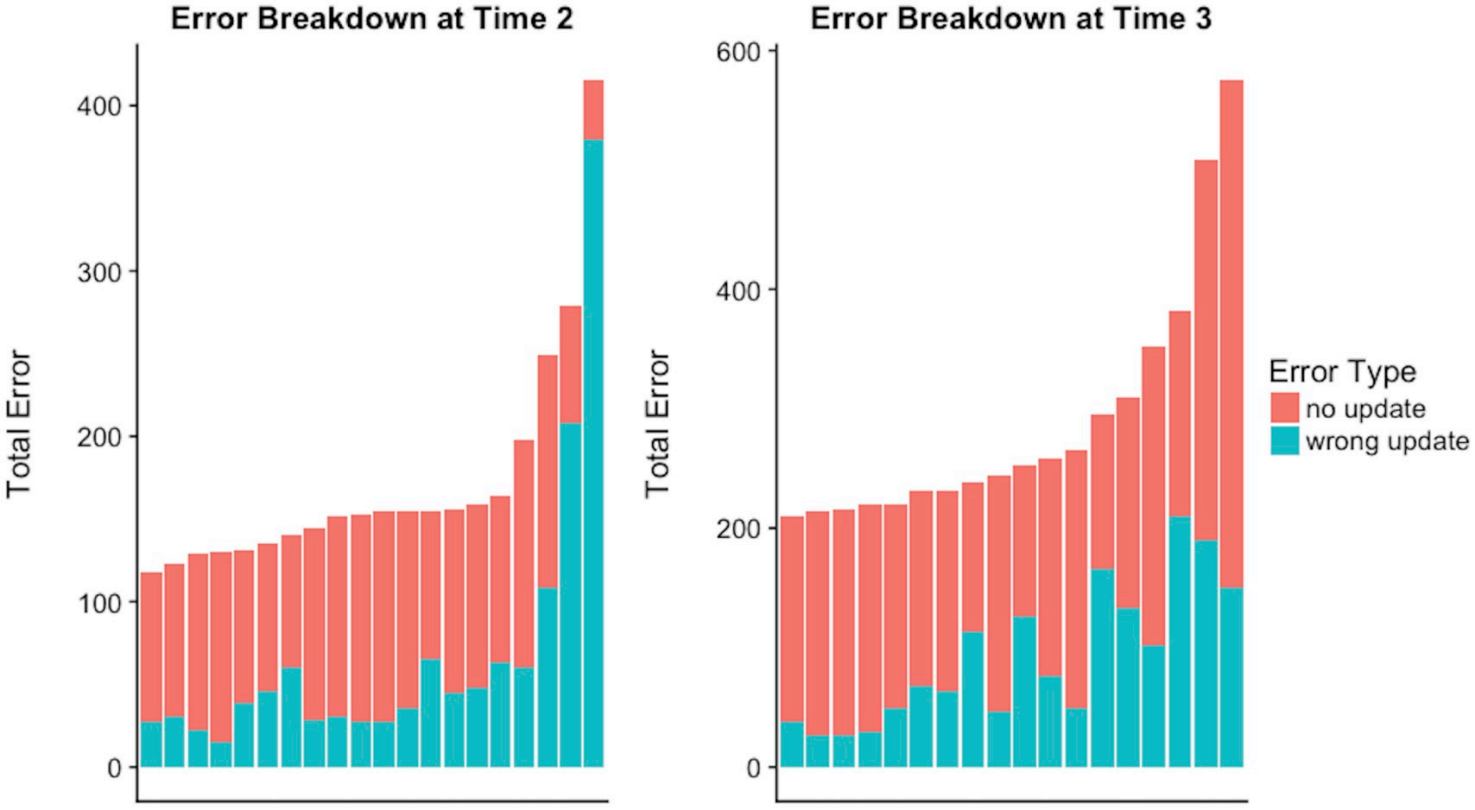
Error breakdown at Time 2 and Time 3 for all students.

Fig 6 displays the breakdown of the two types of errors for each of the respondents. At both time points we see the preponderance of the errors occur because the respondent fails to update a prior belief. Going from Time 1 to Time 2, 67% of the errors were due to a failure to update one’s prior perception. Going from Time 2 to Time 3, a similar pattern was found as 68% of the errors were due to a failure to update. This suggests that perception accuracy of social networks are very much dependent on the perceptions emerging during the initial stages of network formation.

The remainder of the errors are a result of incorrect updates. Incorrect updates can arise either through errors of omission or commission. The data indicate that the majority of incorrect updates from Time 1 to Time 2 (69%) and from Time 2 to Time 3 (59%) are due to errors of commission. However, this is not surprising; due to the low density of many social networks individuals have a much greater chance of perceiving non-existent ties as present then present ties as non-existent. Our finding of higher tendencies to commission errors aligns with prior research on ego-network perception. Krackhardt [45] examined four different data sets and found that errors of commission were much more prevalent than errors of omission for both advice ties and friendship ties. Experimental evidence is needed in order to tease out the exact mechanisms through which path dependency related errors may shape network cognition (e.g., an active search for corroborating information vs. misperceiving available evidence as in confirmation bias).

### Perceiving the arc of friendships

The final component of our analysis moves from examining errors at a given time point to errors occurring in one’s overall perception of the narrative arc or storyline of a given dyad. Rather than assessing the respondent’s accuracy in perceiving whether *i* and *j* were friends at each of the three time points, we consider the arc of their friendship across all three time points and examine whether respondents were able to perceive the friendship storyline correctly. As noted above, given three time points, there are eight distinct storylines that any dyad could follow. These storylines fall into four general categories: stable dyads (dyads that never change friendship status), dissolving friendships (dyads that were friends at earlier time points but not later), emerging friendships (dyads initially not connected but became and stayed friends later), and on-again, off-again (where the dyad switches between friends and not friends at each time point). Table 2 reports the average of correct perception percentages for four storyline categories. For example for each of the 440 stable dyads in the network, we first calculate the percentage of individuals that correctly perceive each stable dyad. We then average those percentages across all stable dyads and report the average percentage correct. For stable dyads this turns out to be 64.8%. We should note that among the stable dyads, about 90% have no relation at all time points and about 10% have relations at all time points. We then calculate the remaining percentages using the same procedure for the other dyad types.

**Table 2:** Perception of storylines.

Storyline	Total Number	Average Correct Perception
Stable Dyads	440	64.8%
Emerging Friendships	80	12.5%
Dissolving Friendships	81	11.1%
On-Again, Off-Again	49	8.2%

Perhaps unsurprisingly, the least accurate perceptions occurred for the on-again, off-again relationships. On average correct perception percentage of on-again, off-again relations were only 8.2%. The averages for emerging friendships and dissolving friendships are 12.5% and 11.1%, respectively. Of all storylines, the stable dyads were by far most accurately perceived with an average correct perception percentage of 64.8%. This may be driven by the fact that people often do not update their prior beliefs, which may be part of the increased accuracy, but it also requires people to accurately judge the existence of friendships at the first time point. It may be that those who are always friends or who are never friends display or demonstrate their affective feelings in a way that is more visible or perceptible to others.

However, the lower levels of accuracy for unstable versus stable friendships may also be driven in part by measurement error. When friendships are very strong, both members of the dyad are easily able to identify the current relationship as a friendship. But when the tie is weak or in flux, it is likely that the respondent is not completely certain of its status and thus more prone to measurement error when asked about that relationship.

## Discussion

Despite the growth in network cognition studies based on CSS designs over the past few decades, empirical research has been limited by the cross-sectional nature of nearly all datasets. In order to address this gap, we engaged in four sets of analyses. First we asked whether or not individuals become more accurate in their perceptions of the network over time. We found no significant increase in accuracy. In fact, as demonstrated in Fig 3, the individual trends in accuracy across time varied considerably. Some students appeared to learn, as demonstrated by an improved accuracy score at each time point. Several students’ accuracy remained relatively stable, whereas others performed worse over time, a few drastically so. This finding highlights the dynamic nature of network cognition, and also raises the question of reliability of network cognition and accuracy studies based on the measurement of accuracy at a single time point. As our findings indicate, an individual with highly accurate network cognition at one time point may have significantly lower accuracy at another time point. Future research may focus on revealing the determinants of heterogeneous trajectories of network cognition over a period of time.

Second, we shifted from exploring one’s cognitive representation of the overall network to examining one’s perception of his or her own direct ties. Individuals can hold an inaccurate view of their direct ties if their claims of friendship to another are not confirmed. Prior research has suggested that humans have an egocentric view of their networks and therefore tend to inflate their own centrality. Examining this bias over time, we find a consistent tendency to claim more incoming friendship ties than one’s peers actually send. No significant changes were found in the amount by which people inflated their popularity over time. However, we did find that individuals were quite capable of recognizing trends in their network position. Using indegree centrality as a measure of popularity, we found that despite a consistent tendency to inflate one’s popularity at any given time point, respondents were quite accurate in identifying changes in their popularity. This suggests that individuals are able to perceive changes in the broader dynamics of social hierarchy even as they become no more accurate in understanding either the overall network or their own ego-net.

Third, we explored perception errors in more detail and analyzed the role that path dependency may play in determining error rates. With longitudinal data, we categorized all of the errors that occurred at Time 2 and Time 3 as having one of two possible origins. The first of these is a failure to update. Here, an individual retains their prior assessment of a dyad, which turns out to be false at the current time point. The second error origin is an incorrect update. Here, the individual changes their prior assessment of a dyad to a status that does not match its current status. At both Time 2 and Time 3, we found that nearly 70% of all errors were due to a failure to update. Thus there was a significant amount of path dependency in individual’s cognitive perceptions, which led to the majority of errors in the data. Hence our study shows well known biases play a role in shaping human perceptions of social structure as well as highlights the dominance of initial network cognition on long term trends.

Finally, we considered the narrative arc of dyadic relations. While inferential network analysis techniques focus on identifying the presence or absence of ties at a particular point in time, we traced the evolution of each relationship across the three time points to assess how well respondents were able to follow the friendship relations of their peers. We found that individuals were only accurate in following stable dyads (i.e., those that were always friends or never friends). For emerging friendships, dissolving friendships, and on-again, off-again relations, the average correct perception percentages were 12.5%, 11.1% and 8.2%, respectively. Therefore, much of the accuracy in people’s ability to perceive their network structure lies in their ability to perceive stable dyads. This may be one reason why we see little change in accuracy from Time 1 to Time 3. As noted above, individuals tend not to update prior assessments of the status of a dyad and therefore may be better able to track stable dyads not simply because they can follow them more easily but because those dyads align with known heuristics and biases.

Overall perception accuracy did not increase for our sample, some respondents did improve at each time point while others did not. As with prior CSS scholarship on the antecedents of accuracy, future longitudinal studies of network perceptions should explore the factors associated with perception improvement and decline. We think several questions are particularly relevant. Why are some relations more visible than others? Are there factors at the individual or dyadic level that allow others to more accurately perceive the status or the storyline of certain ties? Or is accuracy of ties and storylines driven more by biases than visibility? What are the social or economic implications of improvements or declines in network perceptions? For instance, do people who improve their ability to perceive their social surroundings accrue social or professional advantage over those that do not?

While this study offers an initial exploration of longitudinal CSS data, there are several limitations worth noting. Most notably, our data is observational and limited to a single cohort of MBA students. Due to cognitive limitations of recalling large networks, CSS studies, such as ours, use small network settings, which may have an impact on node level statistical tests. While the students in our sample operate in a competitive environment, there are likely to be important differences across social networks based on context. Social network accuracy may be driven by endogenous factors, such as the formal and informal rules, types of tasks, nature of hierarchies, network culture and identity, and spatial arrangements. Hence longitudinal CSS data from varying contexts is needed.

## Supporting information

S1 Appendix (PDF)

S1 Dataset (ZIP)
