# Supplementary material for: Perception accuracy, biases and path dependency in longitudinal social networks
Source: PLoS One. 2019 Jun 18;14(6):e0218607. doi: 10.1371/journal.pone.0218607 (PMC6581280; doi:10.1371/journal.pone.0218607)
Supplement: S1 Appendix — (PDF) [file pone.0218607.s001.pdf]

Supplementary Materials for: "Perception accuracy, biases and path dependency in longitudinal social networks"

Approach to Missing Data: Krackhardt vs. RTM Approach

We carried out a numerical study in order to assess the performance of the RTM method for defining the true network. The RTM method is compared with the approach used by Krackhardt (1990), which defines the true network from CSS objects with missing data as follows: if both individuals constituting the tie are observed use intersection for detecting the true nature of that tie (i.e., LAS Intersection), if only one is observed use it directly (LAS union), if neither members of the dyad are observed qualify the tie as an existing tie only if k (typically $k = n/2$) perceptions are reported by the others.

Table 1 displays a summary of our numerical study, based on Time 1 data after completely removing the three students who did not respond to the survey. Thus, for this comparison we rely on the 23 students who provided their CSS data and thus are able to calculate the "true" network for all dyads from LAS-Intersection. We explore the performance of RTM vs the Krackhardt (1990) method by randomly assigning certain respondent's data to be missing. Each row in the table represents the number of students we assigned to have missing observations (from 1 to 8), followed by the possible number of ways of having the indicated number of missing observations. For example, since $n = 23$, there are C_1^{23}=23 ways of missing just one observation, C_2^{23}=253 ways of missing two observations, etc. In our numerical study, we consider all possible ways of the indicated number of missing observations, define the true network (based on the given missing data) using RTM and Krackhardt's (1990) method, calculate the correlation of these true networks with the actual true network, and report the mean correlation. For further clarification, let us focus on one missing observation cases. First we define the true network using LAS intersection, let us call it t^*.Then we pretend that the first observation is missing, estimate the true network using RTM and Krackhardt's (1990) method, and call them t_1^{RTM} and t_1^{KR}. We repeat this 23 times as there are 23 different ways of having only one missing observation, and record the correlations $cor(t^*, t_i^{RTM})$ and $cor(t^*, t_i^{KR})$ for $i = 1, , 23$. Once we consider all these, we move on to two missing observation cases and record the correlations for 253 possible ways, and proceed in the same fashion until we record all correlations for 8 missing observation cases which can be done in C_8^{23}=$490,314$ different ways. The mean correlations are reported in the related rows of Table 1. Figure 1 displays the distribution of these correlations. It is clear that in case of missing observations using RTM is the more accurate way of defining the true network.

Krackhardt, D. (1990). Assessing the political landscape: Structure, cognition, and power in organizations. *Administrative Science Quarterly*, 35:342-369.

number treated as missing	percentage treated as missing	mean RTM method	stdev RTM method	mean Krackhardt	st dev Krackhardt	# of combinations
1	4	0.9552	0.0184	0.9442	0.0289	23
2	9	0.9124	0.0240	0.8945	0.0372	253
3	13	0.8711	0.0276	0.8427	0.0438	1771
4	17	0.8313	0.0302	0.7922	0.0487	8855
5	22	0.7925	0.0319	0.7506	0.0506	33649
6	26	0.7554	0.0330	0.7150	0.0511	100947
7	30	0.7197	0.0339	0.6666	0.0543	245157
8	35	0.6854	0.0348	0.6184	0.0570	490314

Table 1: RTM vs. Krackhardt

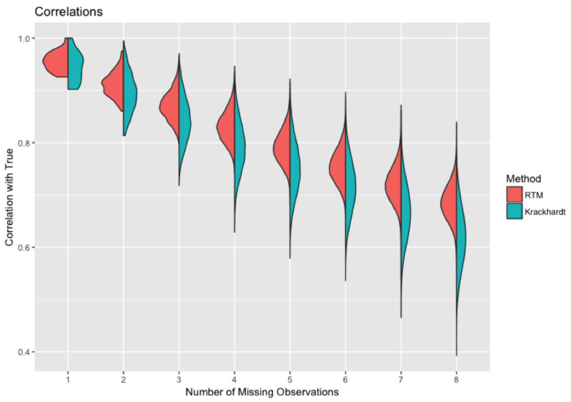

Figure 1: Correlation Distributions, RTM vs. Krackhardt.

Identifying Threshold for Friendship

Students self reported friendship was measured based on a weighted scale (0-Just a fellow student; 1-Slight friends(acquaintance); 2- Fairly good friends; 3-Close friends; 4-Best friends). Any tie reported as 2 or above are considered as friends. Thus, any tie claimed to be stronger than slight friends/acquaintance was coded as a 1 and all ties listed as just a fellow student or an acquaintance were coded as 0. This cut off point is commonly used in the literature (Almaatuq et al., 2016). However, we still wanted to make sure this decision coincides with how the respondents understand friendship. In order to assess this, we take each respondents column in their CSS slice, which represents their claims of incoming friendship ties and use those columns to construct a complete network. In other words, we are using respondents claims of who sends a tie to them to define the network. These are binary claims of incoming ties, and thus reflect the level of friendship the respondents deem necessary to classify one as a friend. We then calculated the density of that network, which is in the last column of the table below. The density is .349. When comparing the density of the network based on incoming ties to the density of the true network when using different weights for their outgoing ties (i.e., the respondents row in the CSS network) we see the closest correspondence is at our current approach of using fairly food friends and above. As expected, when the cut-point for determining a tie is low, in this case when allowing slight friends (acquaintance) to be considered a friend,

	Density Cut 1 (slight friends)	Density Cut 2 (fairly good friends)	Density Cut 3 (close friends and best friends	Column Density
Time 1	0.62	0.38	0.13	0.35

Table 2: Density of true networks with different cuts vs. column density

the density of the resulting network is extremely high, over .61. If we make the requirement for a friend too stringent, in this case only close friends and above, the density is rather low, just .13. Using our current procedure of labeling ties at fairly good friends and above as 1s, we find the density of the network based on incoming ties matches our observed density. This lends additional support to our method to binarize the self-reported ties.

We also checked whether perception accuracy improves over time when different thresholds are used. As expected, average perception accuracy increases as criterion for having a friendship tie becomes more strict. However, when we consider if accuracy scores increase from Time 1 to Time 2, the finding that accuracy does not improve over time holds true across different thresholds. (Threshold slight friends and above: Time 1: Mean = 0.33 and SD =0.08 and Time 2: Mean = 0.34 and SD = 0.08; Threshold fairly good friends and above-(threshold used in the paper): Time 1: Mean = 0.37 and SD = 0.86 and Time 2: Mean = 0.36 and SD = 0.09; Threshold close friends and above: Time 1: Mean-0.43 and SD-0.09 and Time 2: Mean = 0.43 and SD = 0.15; and Threshold best friends: Time 1: Mean = 0.43 and SD = 0.09 and Time 2: Mean = 0.43 and SD = 0.15).

Almaatouq A, Radaelli L, Pentland A, Shmueli E (2016) Are You Your Friends Friend? Poor Perception of Friendship Ties Limits the Ability to Promote Behavioral Change. *PLoS ONE* 11(3): e0151588.
